# Functional Characterisation of Three *O*-methyltransferases Involved in the Biosynthesis of Phenolglycolipids in *Mycobacterium tuberculosis*

**DOI:** 10.1371/journal.pone.0058954

**Published:** 2013-03-11

**Authors:** Roxane Simeone, Gaëlle Huet, Patricia Constant, Wladimir Malaga, Anne Lemassu, Françoise Laval, Mamadou Daffé, Christophe Guilhot, Christian Chalut

**Affiliations:** 1 Centre national de la recherche scientifique, Institut de Pharmacologie et de Biologie Structurale, Toulouse, France; 2 Université de Toulouse, Université Paul Sabatier, Institut de Pharmacologie et de Biologie Structurale, Toulouse, France; University of Helsinki, Finland

## Abstract

Phenolic glycolipids are produced by a very limited number of slow-growing mycobacterial species, most of which are pathogen for humans. In *Mycobacterium tuberculosis*, the etiologic agent of tuberculosis, these molecules play a role in the pathogenicity by modulating the host immune response during infection. The major variant of phenolic glycolipids produced by *M. tuberculosis*, named PGL-tb, consists of a large lipid core terminated by a glycosylated aromatic nucleus. The carbohydrate part is composed of three sugar residues, two rhamnosyl units and a terminal fucosyl residue, which is per-*O*-methylated, and seems to be important for pathogenicity. While most of the genes responsible for the synthesis of the lipid core domain and the saccharide appendage of PGL-tb have been characterized, the enzymes involved in the *O*-methylation of the fucosyl residue of PGL-tb remain unknown. In this study we report the identification and characterization of the methyltransferases required for the *O*-methylation of the terminal fucosyl residue of PGL-tb. These enzymes are encoded by genes *Rv2954c*, *Rv2955c* and *Rv2956*. Mutants of *M. tuberculosis* harboring deletion within these genes were constructed. Purification and analysis of the phenolglycolipids produced by these strains, using a combination of mass spectrometry and NMR spectroscopy, revealed that *Rv2954c*, *Rv2955c* and *Rv2956* encode the methyltransferases that respectively catalysed the *O*-methylation of the hydroxyl groups located at positions 3, 4 and 2 of the terminal fucosyl residue of PGL-tb. Our data also suggest that methylation at these positions is a sequential process, starting with position 2, followed by positions 4 and 3.

## Introduction

**Figure 1:**
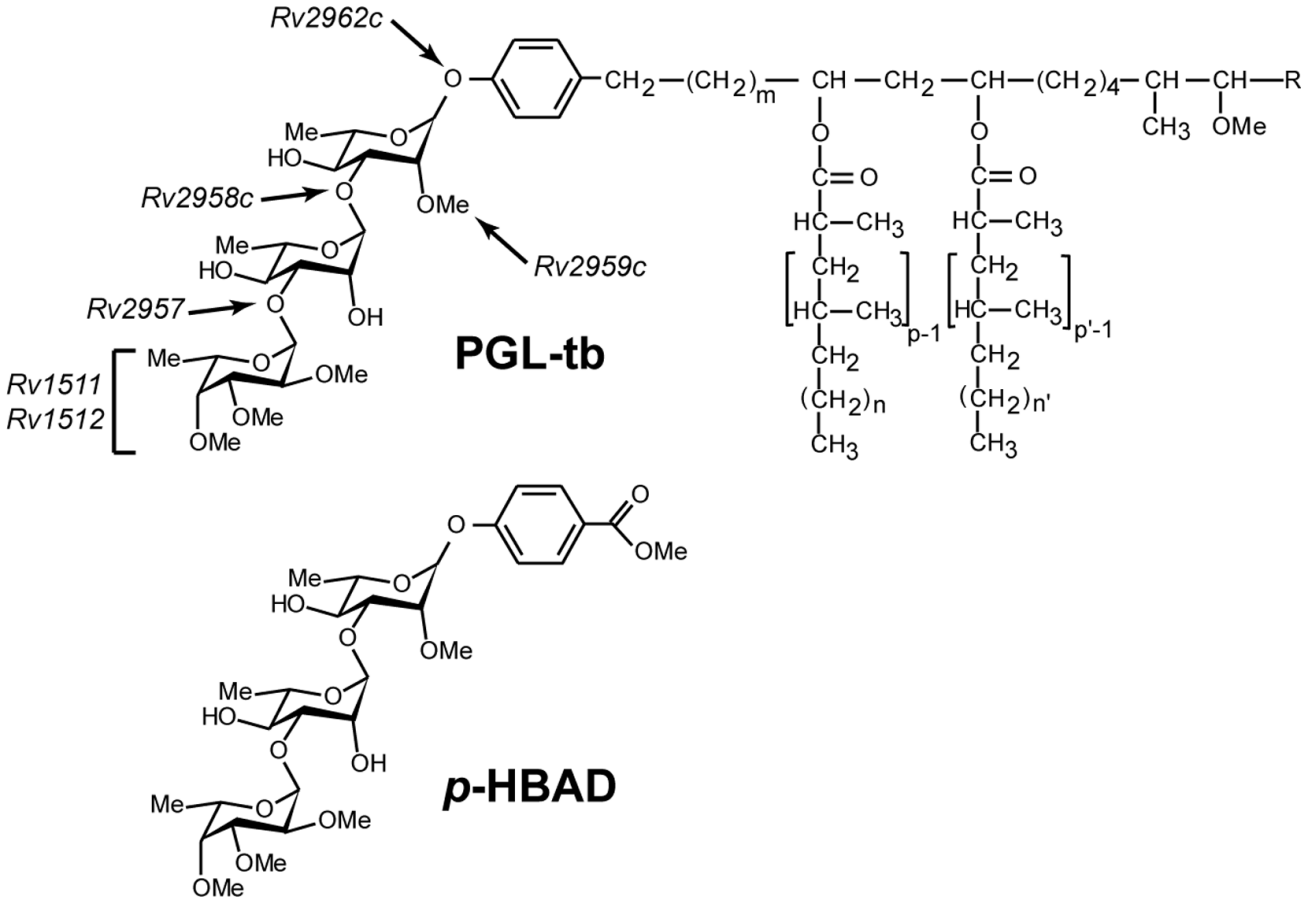
Structure of PGL-tb and of *p*-HBAD produced by *M. tuberculosis*. The genes involved in the synthesis of the carbohydrate moiety of PGL-tb are indicated. m = 15–17; n, n’ = 16–18; p, p’ = 2–4.

Phenolic glycolipids (PGL) are found in a limited group of pathogenic mycobacteria including the members of the *M. tuberculosis* complex, *M. leprae*, and a few other slow-growing mycobacteria (*M. kansasii, M. gastri*, *M. ulcerans, M. marinum*, *M. microti* and *M. haemophilum*) [1], [2]. These substances, located in the outermost layer of the mycobacterial cell envelope, have been shown to play important functions in the pathogenicity of these bacteria [3], [4], [5], [6]. PGL are composed of a mixture of long chain β-diols, esterified by multimethyl-branched fatty acids, named mycocerosic acids or phthioceranic acids depending on the configuration of the asymmetric centres bearing the methyl branches [1]. The β-diols chain is terminated by an aromatic nucleus, which in turn is glycosylated (Figure 1). The sugar moiety of PGL consists of 1 to 4 sugar residues, depending on the mycobacterial species, and most are *O*-methylated deoxysugars [2]. In *M. tuberculosis* the carbohydrate domain of the major variant of PGL, named PGL-tb, is 2,3,4-tri-*O*-Me-α-L-Fuc*p*(1→3)-α-L-Rha*p*(1→3)-2-*O*-Me-α-L-Rha*p*(1→) (Figure 1). *M. tuberculosis* also secretes a family of smaller molecules that contain the same glycosylated phenolic moiety as PGL-tb, the glycosylated *p*-hydroxybenzoic acid methyl esters (*p*-HBAD) (Figure 1) [7].

Although PGL-tb are absent from many *M. tuberculosis* strains, due to a natural frameshift mutation within *pks15/1*, a gene encoding a type-I polyketide synthase specifically involved in the biosynthesis of PGL-tb [7], several lines of evidence indicate that they may contribute to the pathogenesis of *M. tuberculosis*. For instance, it was reported that a *M. tuberculosis* strain producing PGL-tb induced more severe tuberculous meningitis in infected rabbits than did strains devoid of PGL-tb [5]. Moreover, the production of PGL-tb in one *M. tuberculosis* isolate was associated with a hypervirulence phenotype in the mouse model [4]. This observation was correlated with the finding that PGL-tb inhibits the production of several pro-inflammatory cytokines by infected mouse macrophages. This phenotype was dependent on the structure of the saccharide moiety of PGL-tb since the structurally related monoglycosylated PGL produced by *M. bovis* BCG, the so-called mycoside B, did not produce a similar effect [4]. The putative role of the saccharide moiety in the immunomodulation activity of PGL-tb was further highlighted by the finding that *p*-HBAD inhibits the pro-inflammatory response of infected macrophages [8]. Thus it appears that the carbohydrate moiety of PGL-tb and of *p*-HBAD likely plays an important role in the pathogenesis of mycobacterial infections.

**Figure 2:**
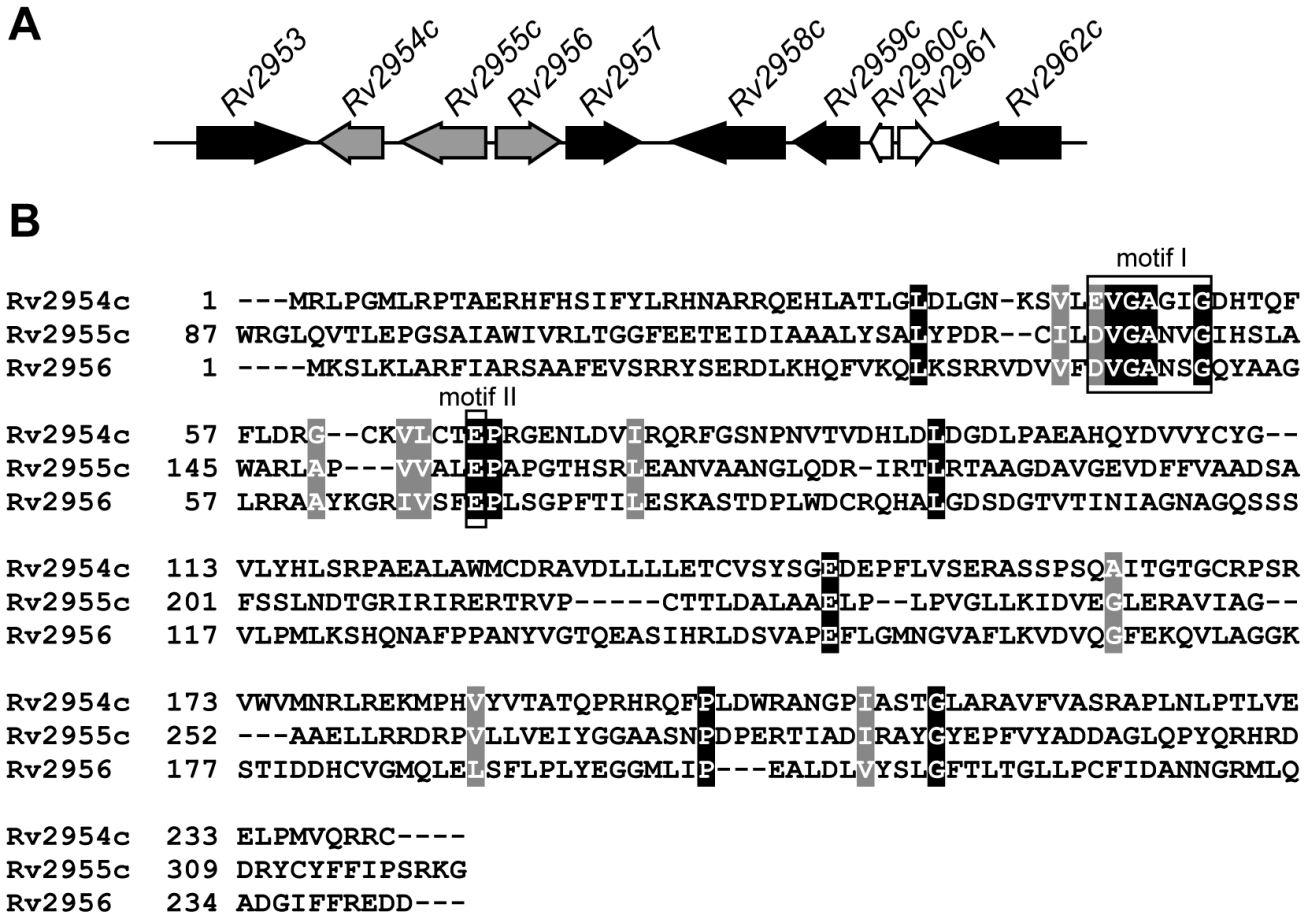
Localization of *Rv2954c*, *Rv2955c*, and *Rv2956* within the DIM+PGL locus and multiple alignment of the amino acid sequences predicted to be encoded by these genes. (**A**) Genetic organisation of the DIM+PGL locus in *M. tuberculosis* from *Rv2953* to *Rv2962c*. Genes belonging to the PGL-tb synthesis pathway are represented by solid black arrows and *Rv2954c*, *Rv2955c*, *Rv2956* by solid gray arrows. *Rv2960c* and *Rv2961* (open arrows) encode a hypothetical unknown protein and a putative transposase, respectively, and are probably not involved in PGL biosynthesis. (**B**) Multiple sequence alignment based on the primary sequences of Rv2954c (residues 1–241), Rv2955c (residues 87–321) and Rv2956 (residues 1–243). Alignment was performed using the CLUSTALW algorithm (EMBnet, http://www.ch.embnet.org). Motifs I and II found in SAM-methyltransferases are boxed.

The genes involved in the biosynthesis of the lipid core of PGL-tb are clustered on a 70 kb region of the mycobacterial chromosome, the so-called DIM+PGL locus [9], [10]. This chromosomal region also contains three glycosyltransferase-encoding genes, namely *Rv2957*, *Rv2958c* and *Rv2962c*, and another gene (*Rv2959c*) encoding a methyltransferase required for the formation of the carbohydrate part of PGL-tb and *p*-HBAD (Figures 1 and 2A). Two additional genes (*Rv1511* and *Rv1512*) located outside of the DIM+PGL locus and responsible for the formation of L-fucose in *M. tuberculosis*, have been shown to be involved in the biosynthesis of the terminal fucosyl residue of PGL-tb [11]. Despite these advances, the genes involved in the *O*-methylations of the fucosyl residue of PGL-tb remain uncharacterized. In this study, we report the identification of the three methyltransferases responsible for modifying the hydroxyl groups at positions 2, 3, and 4 of the terminal fucosyl unit of PGL-tb.

## Materials and Methods

### Bacterial Strains, Growth Media and Culture Conditions

Plasmids were propagated at 37°C in *E. coli* DH5α or HB101 in LB broth or LB agar (Invitrogen) supplemented with either kanamycin (Km) (40 µg/ml) or hygromycin (Hyg) (200 µg/ml). The *M. tuberculosis* H37Rv Pasteur wild-type strain and its derivatives were grown at 37°C in Middlebrook 7H9 broth (Difco) containing ADC (0.2% dextrose, 0.5% bovine serum albumin fraction V, 0.0003% beef catalase) and 0.05% Tween 80 when necessary and on solid Middlebrook 7H11 broth containing ADC and 0.005% oleic acid (OADC). For biochemical analyses, mycobacterial strains were grown as surface pellicles on Sauton’s medium. When required, Km and Hyg were used at a concentration of 40 µg/ml and 50 µg/ml, respectively. Sucrose 2% (w/v) was used to supplement 7H11 for the construction of the PMM115 and PMM116 mutants.

### General DNA Techniques

Molecular cloning experiments were performed using standard procedures. Mycobacterial genomic DNA was extracted from 5 ml saturated cultures as previously described [12]. PCR experiments for plasmid construction or genomic analysis were performed in standard conditions on a GeneAmp PCR system 2700 thermocycler (Applied Biosystem). PCR was performed in a final volume of 50 µl containing 2.5 units of Pfu DNA polymerase (Promega).

### Construction of *M. tuberculosis* H37Rv Gene-disrupted Mutants

The Δ*Rv2954c* and Δ*Rv2956 M. tuberculosis* H37Rv mutants (PMM115 and PMM116, Table 1) were constructed by allelic exchange using the Ts/*sacB* procedure [13]. Two DNA fragments containing either the *Rv2954c* gene or the *Rv2956* gene were amplified by PCR from *M. tuberculosis* genomic DNA using oligonucleotides 2954A and 2954B (for *Rv2954c*) and 2956A and 2956B (for *Rv2956*) (Table 2) and subsequently cloned into pGEM-T (Promega). An internal 463 bp *Rv2954c* fragment and an internal 487 bp *Rv2956* fragment were removed by double-strand site-directed mutagenesis with the inverse PCR technique using oligonucleotides 2954IP1 and 2954IP2 for *Rv2954c* and oligonucleotides 2956IP1 and 2956IP2 for *Rv2956* and substituted by a *km* resistance cassette formed by the Ω*km* cassette flanked by two *res* sites from transposon γδ [14]. The DNA fragments containing the disrupted *Rv2954c* and *Rv2956* genes were inserted into pPR27, a mycobacterial thermosensitive suicide plasmid harboring the counterselectable marker *sacB* and the resulting plasmids were transferred by electrotransformation into *M. tuberculosis* for allelic exchange. PCR screening for disruption of *Rv2954c* and *Rv2956* were performed with a set of specific primers (2954C, 2954D, 2954E, res1, and res2 for *Rv2954c* and 2956C, 2956D, 2956E, res1, and res2 for *Rv2956*, Table 2 and Figure S1) after extraction of the genomic DNA from several Km and sucrose-resistant colonies. Two clones giving the expected pattern for disruption of *Rv2954c* and *Rv2956* were selected for further analyses and named PMM115 (*Rv2954c::res-km-res*) and PMM116 (*Rv2956::res-km-res*), respectively.

**Table 1:** Strains and plasmids.

Name	Relevant characteristics	Ref./source
Strain		
PMM115	*M. tuberculosis* H37Rv Δ*Rv2954c::res-km-res*, Km^R^	This study
PMM144	*M. tuberculosis* H37Rv Δ*Rv2954c::res*	This study
PMM126	*M. tuberculosis* H37Rv Δ*Rv2955c::res-km-res*, Km^R^	This study
PMM145	*M. tuberculosis* H37Rv Δ*Rv2955c::res*	This study
PMM116	*M. tuberculosis* H37Rv Δ*Rv2956::res-km-res*, Km^R^	This study
PMM122	*M. tuberculosis* H37Rv Δ*Rv2956::res*	This study
Plasmid		
pPET1	pMIP12H containing *pks15/1* from *M. bovis* BCG, Hyg^R^	[7]
pRS18	pMV361e containing *Rv2954c* from *M. tuberculosis*, Km^R^	This study
pRS19	pMV361e containing *Rv2955c* from *M. tuberculosis*, Km^R^	This study
pRS26	pMV361e containing *Rv2957c* from *M. tuberculosis*, Km^R^	This study
pRS27	pMV361e containing *Rv2956* and *Rv2957c* from *M. tuberculosis*, Km^R^	This study

**Table 2:** Oligonucleotides used in this study.

Gene	Primer	Oligonucleotide sequence 5′–3′
*Rv2954c*	2954A	AGGTTTAAACCTCCAGGTCACCCTTGAGC
	2954B	AGGTTTAAACACCCGAACTTGGTGCGCAGC
	2954C	GGATGTTAGAGTGCGCCATGC
	2954D	CGTACACGCTGACCACGGAC
	2954E	GGAAGAACTGCGTGTGATCGC
	2954F	ACAACATATGCGACTCCCCGGCATGTT
	2954G	ACACAAGCTTCAGCACCGGCGCTGCAC
	2954IP1	ATCGACTGAGGGAGAAGATGCC
	2954IP2	ATCTGCCGAAGGTAAAAGATGCTG
*Rv2955c*	2955H	GGTTTAAACGCATGCCACCTTCGTACAACG
	2955I	GGTTTAAACCGAGGAAGCCCTCTCACTAAC
	2955J	AAGGCAGCAATCCCGTCAACG
	2955K	CTCAGTCGGTTCATCACCCAC
	2955L	TCGAGGATGCAGCGGTCTGG
	2955F	ACAACATATGCAGTTCCAAGATGTGCGC
	2955G	ACACAAGCTTCTATCCCTTCCGGCTCGGAA
*Rv2956*	2956A	AGGTTTAAACGTTTAGCGAACTGAAGGCGC
	2956B	AGGTTTAAACACGGTGCGCATTACCATGGC
	2956C	CGTCCAGCGTGGTACACGG
	2956D	GTCAACGTTGCGGTGGTATCG
	2956E	ATGGGCAAGACGGAACTGCTC
	2956F	ACAACATATGCAGTTCCAAGATGTGCGC
	2956IP1	GAGTAATGGTCGAATGTTGCAGGC
	2956IP2	GAGGCCCTTATATGCTGCTCGG
*Rv2957*	2957A	ACAACATATGGTGCAGACGAAACGATAC
	2957B	ACACAAGCTTGCGGTGGTATCGCGCTAAC
*res*	res1	GCTCTAGAGCAACCGTCCGAAATATTATAAA
	res2	GCTCTAGATCTCATAAAAATGTATCCTAAATCAAATATC

To construct a *Rv2955c M. tuberculosis* mutant of H37Rv, we used the strategy described by Bardarov *et al.* [15]. A 2369 bp fragment containing the *Rv2955c* gene was amplified from *M. tuberculosis* genomic DNA using primers 2955H and 2955I (Table 2) and inserted within the vector pGEM-T. The *km* resistance cassette was then inserted between the *Bst*EII and *Eco*RI sites of the *Rv2955c* gene and the fragment containing the *Rv2955c* disrupted allele was cloned within the cosmid vector pYUB854 [15]. The resulting cosmid, was cut with *Pac*I and ligated with the mycobacteriophage phAE87. The ligation products were encapsidated *in vitro* using the Gigapack III XL kit (Stratagene) and the mix was used to infect *E. coli* HB101 following the manufacturer recommendations. Transfectants were selected on LB plates containing Km. A recombinant phagemid containing the disrupted gene construct was selected and transferred by electroporation in *M. smegmatis* and phage particules were prepared as described previously [15]. These particles were then used to infect *M. tuberculosis* H37Rv and *M. tuberculosis* allelic exchange mutants were selected by PCR analysis using primers 2955J, 2955K, 2955L, res1 and res2 (Table 2 and Figure S1). One clone, named PMM126 (*Rv2955c::res-km-res*), gave the pattern corresponding to allelic exchange and was retained for further analysis.

The recovery of the *res*-Ω*km-res* cassette from *M. tuberculosis* PMM115, PMM116 and PMM126 was performed as previously described using the thermosensitive plasmid pWM19 that contains the resolvase gene of transposon γδ [14]. Several clones were selected and analyzed by PCR using various primers (2954C and 2954D for *Rv2954c*, 2955J and 2955K for *Rv2955c*, and 2956C and 2956D for *Rv2956*, Figure S1). Three clones giving the expected pattern for excision of the *res*-Ω*km-res* cassette in *Rv2954c*, *Rv2955c* and *Rv2956*, were selected and named PMM144 (*Rv2954c::res*), PMM145 (*Rv2955c::res*) and PMM122 (*Rv2956::res*), respectively.

### Construction of Complementation Plasmids

To construct pRS18 and pRS19, a region covering the *Rv2954c* gene and a region covering the *Rv2955c* gene were PCR-amplified from *M. tuberculosis* H37Rv genomic DNA using oligonucleotides 2954F and 2954G (for *Rv2954c*) and 2955F and 2955G (for *Rv2955c*) (Table 2). The PCR products were digested with *Nde*I and *Hind*III endonuclease restriction enzymes and cloned between the *Nde*I and *Hin*dIII sites of pMV361e, a pMV361 derivative containing the pblaF* promoter instead of the original *phsp60* promoter and carrying a *km* resistance marker [16]. For the construction of complement plasmids pRS26 and pRS27, two DNA fragments overlapping either the *Rv2957* (pRS26) gene or the *Rv2956* and *Rv2957* genes (pRS27) were amplified by PCR from *M. tuberculosis* H37Rv genomic DNA using oligonucleotides 2957A and 2957B or 2956F and 2957B (Table 2) and cloned between the *Nde*I and *Hind*III sites of pMV361e.

### Extraction and Purification of Glycolipids

Mycobacterial cells obtained from culture on Sauton’s medium were left in 60 ml of CHCl_3_/CH_3_OH (1∶2, v/v) for 48 h to kill bacteria. Lipids were then extracted twice with CHCl_3_/CH_3_OH (2∶1, v/v) for 24 h each, washed twice with water, and dried. Extracted mycobacterial lipids were suspended in CHCl_3_ at a final concentration of 20 mg/ml and analyzed by thin-layer chromatography (TLC). Equivalent amounts of lipids from each strain were spotted on silica gel G60 plates (20×20 cm, Merck) and separated with CHCl_3_/CH_3_OH (95∶5, v/v). Glycolipids were visualized by spraying the plates with a 0.2% anthrone solution (w/v) in concentrated H_2_SO_4_, followed by heating. Crude lipid extracts were subjected to chromatography on a Sep-Pak Florisil cartridge and eluted with a series of concentrations of CH_3_OH (0, 10, 20, 30%) in CHCl_3_. Each fraction was analyzed by TLC on Silica Gel G60 using CHCl_3_/CH_3_OH (90∶10, v/v) as the solvent system and glycolipids were visualized as described above. For MALDI-TOF mass spectrometry analyses, glycolipids were additionally purified by preparative chromatography on silica gel G60 plates using CHCl_3_/CH_3_OH (90∶10, v/v) as the developing solvent and recovered by scraping silica gel from the plates.

### Matrix-assisted Laser Desorption-Ionization Time-of-Flight (MALDI-TOF) Mass Spectrometry

MALDI-TOF mass spectrometry analyses were performed in reflectron mode, with an Applied Biosystems 4700 analyzer mass spectrometer (Applied Biosystems, Framingham) equipped with an Nd:YAG laser (wawe-length 355 nm; pulse<500 ps; repetition rate 200 Hz). A total of 2500 shots were accumulated in positive ion mode, and mass spectrometry data were acquired with the default calibration for the instrument.

### Nuclear Magnetic Resonance (NMR) Spectroscopy

NMR spectroscopy experiments were carried out at 300° K on a Bruker AVANCE spectrometer operating at 600,13 MHz with a 5-mm triple resonance TCI ^1^H ^13^C ^15^N pulsed field z-gradient cryoprobe. Samples were dissolved in 99,9% CDCl_3_. ^1^H NMR studies on the native and the per-*O*-acetylated glycolipids were performed using one-dimensional and two-dimensional chemical shift correlation spectroscopy (COSY). Chemical shifts are expressed in ppm using chloroform signal as an internal reference (7.265 ppm).

## Results

### *Rv2954c, Rv2955c* and *Rv2956* are Involved in the Biosynthesis of PGL-tb in *M. tuberculosis*

In an attempt to identify the protein(s) responsible for the *O*-methylation of the fucosyl residue in PGL-tb, we searched the *M. tuberculosis* genome for genes encoding methyltransferases and mapping within the DIM+PGL locus. We identified three genes (*Rv2954c*, *Rv2955c* and *Rv2956*) encoding proteins sharing similarities to S-adenosylmethionine (SAM)-dependent methyltransferases. These genes are located in the same chromosomal region as those previously shown to be involved in the synthesis of the sugar moiety of PGL-tb (Figure 2A). Proteins encoded by *Rv2954c* and *Rv2956* are similar in length with 241 and 243 residues, respectively, whereas *Rv2955c* encodes a protein of 321 amino acids. Although their amino acid sequences share little identity (about 15%) (Figure 2B), secondary structure prediction analyses revealed that these proteins are composed of alternative α-helices and β-sheets that are characteristic to the SAM methyltransferase core fold (data not shown). In addition a glycine-rich sequence, close to the E/DXGXGXG motif (motif I) found in a number of SAM-methyltransferases and an acidic region (motif II) located 17–19 amino acid residues downstream from the end of motif I are present in the sequence of each protein (Figure 2B) [17]. Interestingly, motif I starts at position 45 for Rv2954c and Rv2956 but is located at position 132 for Rv2955c suggesting that the latter protein has an additional N-terminal domain.

**Figure 3:**
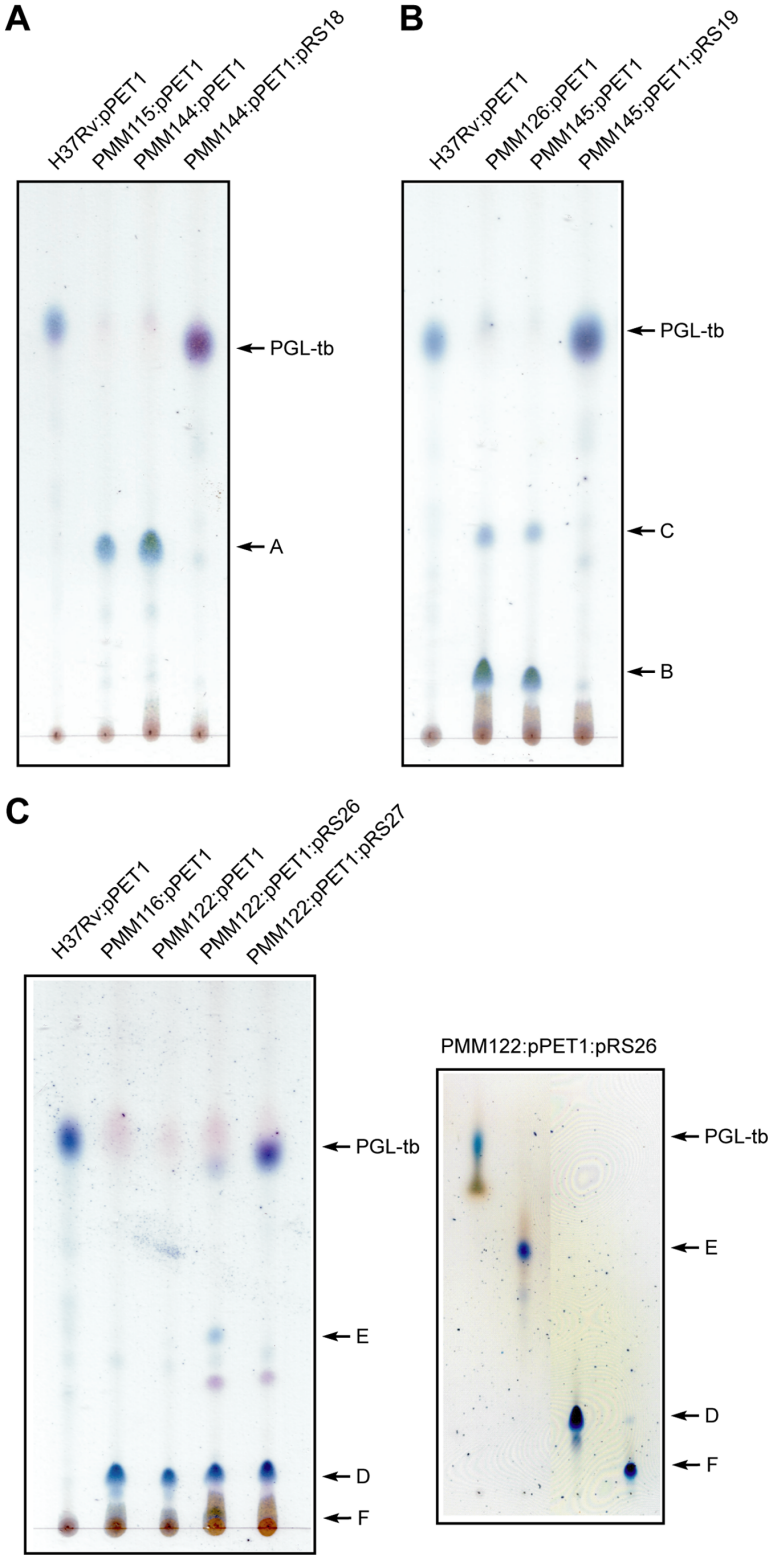
TLC analyses of glycolipids extracted from the wild-type, mutant, and complemented mutant strains of *M. tuberculosis.* Lipids extracted from the *Rv2954c* (**A**), *Rv2955c* (**B**) and *Rv2956* (**C, left panel**) *M. tuberculosis* recombinant strains complemented with pPET1 and from the complemented *M. tuberculosis* mutant strains were spotted on silica gel G60 plates and separated with CHCl_3_/CH_3_OH (95∶5, v/v). Glycolipids were visualized by spraying the plates with a 0.2% anthrone solution (w/v) in concentrated H_2_SO_4_, followed by heating. The positions of PGL-tb and of compounds A, B, C, D, E, F are indicated. Glycolipids from PMM122:pPET1:pRS26 were separated using a Sep-Pak Florisil cartridge and further purified by preparative chromatography on Silica Gel G60. Purified glycoconjugates were spotted on a silica gel G60 plate and separated with CHCl_3_/CH_3_OH (90∶10, v/v) before visualization with anthrone (**C, right panel**).

To investigate the role of these putative methyltransferases in the biosynthesis of PGL-tb, we constructed three *M. tuberculosis* knock-out mutants with insertion within *Rv2954c*, *Rv2955c* and *Rv2956*. These genes were disrupted by insertion of a *km* resistance gene cassette flanked by two *res* sites to yield three recombinant strains, named PMM115 (*Rv2954c::res-km-res*), PMM126 (*Rv2955c::res-km-res*) and PMM116 (*Rv2956::res-km-res*) (Table 1, Figure S1). Since *M. tuberculosis* H37Rv and derivatives are naturally deficient in the production of PGL-tb due to a mutation in the *pks15/1* gene, the mutant strains and the H37Rv wild-type strain were transformed with plasmid pPET1 that carries a functional *M. bovis* BCG *pks15/1* gene [7]. Lipids were extracted from the resulting strains and analyzed by TLC. These analyses showed that the production of PGL-tb was affected in the three mutant strains (Figure 3). The PMM115:pPET1 mutant produced a glycolipid (product A) that exhibits a relative mobility lower than that of PGL-tb. The PMM126:pPET1 mutant produced a major (product B) and a minor (product C) glycoconjugates, both exhibiting a lower mobility than that of PGL-tb. Finally the lipid profile of the PMM116:pPET1 recombinant strain contained a single spot (product D) with a lower mobility than that of PGL-tb. These data suggested that *Rv2954c*, *Rv2955c* and *Rv2956* are involved and play different roles in PGL-tb biosynthesis.

### Rv2954c Catalyses the *O*-methylation of Position 3 of the Fucosyl Residue of PGL-tb

**Figure 4:**
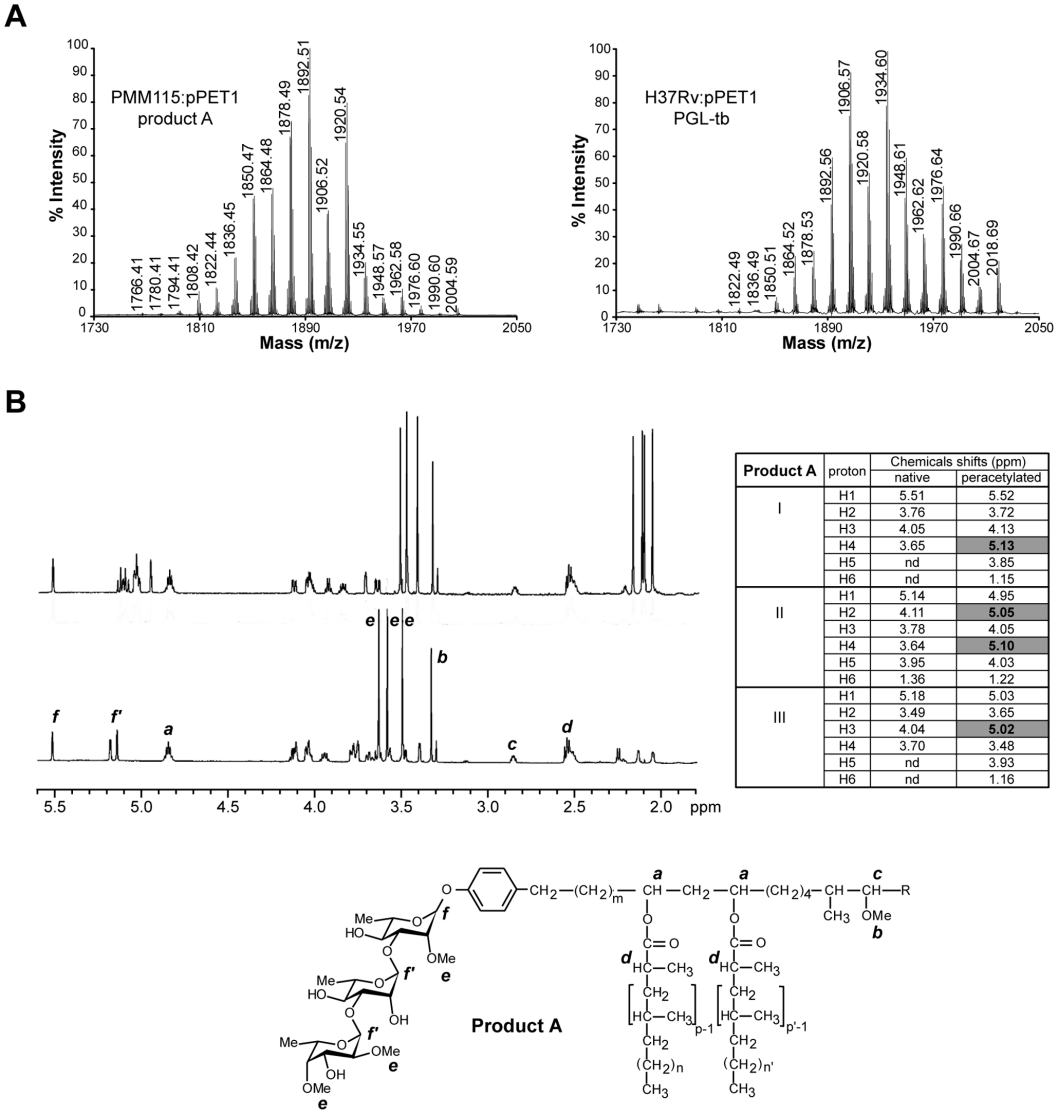
Biochemical analysis of product A from the PMM115:pPET1 mutant. (**A**) MALDI-TOF mass spectra of purified product A from the PMM115:pPET1 mutant strain (left panel) and of purified PGL-tb from *M. tuberculosis* wild-type H37Rv:pPET1 (right panel). (**B**) NMR analysis of product A. 1D ^1^H-NMR spectra (1.8–5.6 ppm) of native (bottom) or per-*O*-acetylated (upper) glycolipid A (600 MHz, in CDCl_3_). The structure of the analyzed compound is shown below the spectrum and the protons corresponding to the main signals are indicated. The table summarizes the assignments of resonances on the basis of chemical shift correlations deduced from the 2D-COSY spectra of native and per-*O*-acetylated compound A. Proton resonances shifted by acetylation are written in bold (grey cells). nd, not determined. Roman numbers in the first column of the table indicate the sugar residue: I, first rhamnosyl residue; II, second rhamnosyl residue; III, terminal fucosyl residue.

To determine the nature of product A produced by the PMM115:pPET1 mutant strain, theglycolipid was purified and analyzed by MALDI-TOF mass spectrometry and ^1^H-NMR. The MALDI-TOF mass spectrum of product A showed a series of major pseudomolecular ion (*M*+Na^+^) peaks at *m/z* 1850, 1864, 1878, 1892, 1906 and 1920, 14 mass units lower than those observed with PGL-tb from H37Rv:pPET1 (Figure 4A) suggesting that this compound may differ from PGL-tb by the absence of one methyl group. As expected, data from one dimensional (1D) ^1^H-NMR analysis of compound A (Figure 4B) were in agreement with the hypothesis. Compared to the NMR spectrum of the native PGL-tb [11], only three signals (3.4–3.7 ppm), instead of four, attributable to the proton resonances of methoxyl groups linked to the sugar moiety were seen in the spectrum of compound A, confirming the loss of one methoxyl group in compound A (Figure 4B). The other NMR data are consistent with the proposed structure, notably (i) the presence of a *p*-substituted phenolic nucleus (two doublets centered at δ = 6.9–7.1); (ii) polymethyl-branched fatty acids (1.14 ppm) esterifying a β-glycol (4.83 ppm); (iii) a methoxyl group borne by the aliphatic chain (3.32 ppm) that characterize a phenolphthiocerol structure. Finally, the presence of three deshielded anomeric protons (5.1–5.6 ppm) confirmed the occurrence of a trisaccharide in the glycolipid. That compound A lacks a methoxyl group was also supported by the occurrence, upon per-*O*-acetylation of four distinct methyl groups of acetyl substituents (signal resonances at 1.9–2.2 ppm), indicating the presence of four non-substituted hydroxyl groups in the native compound A. Moreover, the per-*O*-acetylation resulted in a shift of the resonance of protons linked to the carbons bearing the acetyl group downfield by about 1.0–1.4 ppm; it was thus possible to distinguish free from substituted hydroxyl groups. 2D-COSY NMR analyses of native and per-*O*-acetylated compound A were performed to assign the resonances (Table in Figure 4B). On the basis of chemical shift correlations, we established that it was the position 3 of the fucosyl residue that was per-*O*-acetylated, indicating that this position was free in the native compound A. This suggested that Rv2954c is involved in the methylation of this position.

To firmly establish that production of product A relies to the disruption of the *Rv2954c* gene, we performed gene complementation studies by transferring a wild-type allele of *Rv2954c* into the mutant strain. Due to the limited number of antibiotic resistance genes available for use in mycobacteria, the *res-km-res* cassette was first recovered from the PMM115 strain by site-specific recombination between the two *res* sites [14]. A new strain, named PMM144 (*Rv2954c::res*) harboring only a *res* site at the *Rv2954c* locus (Figure S1) was generated. This strain was transformed either with plasmid pPET1 or with both pPET1 and pRS18 carrying a wild-type allele of *Rv2954c*. TLC and MALDI-TOF analyses revealed that the PMM144:pPET1 strain produced the same PGL-like substance (product A) than PMM115:pPET1 (Figures 3 and S2) whereas the production of PGL-tb was restored in the PMM144:pPET1:pRS18 strain (Figures 3 and S2). Altogether these data established that *Rv2954c* is involved in the *O*-methylation of position 3 of the fucosyl residue of PGL-tb in *M. tuberculosis*.

### Rv2955c Catalyses the *O*-methylation of Position 4 of the Fucosyl Residue of PGL-tb

**Figure 5:**
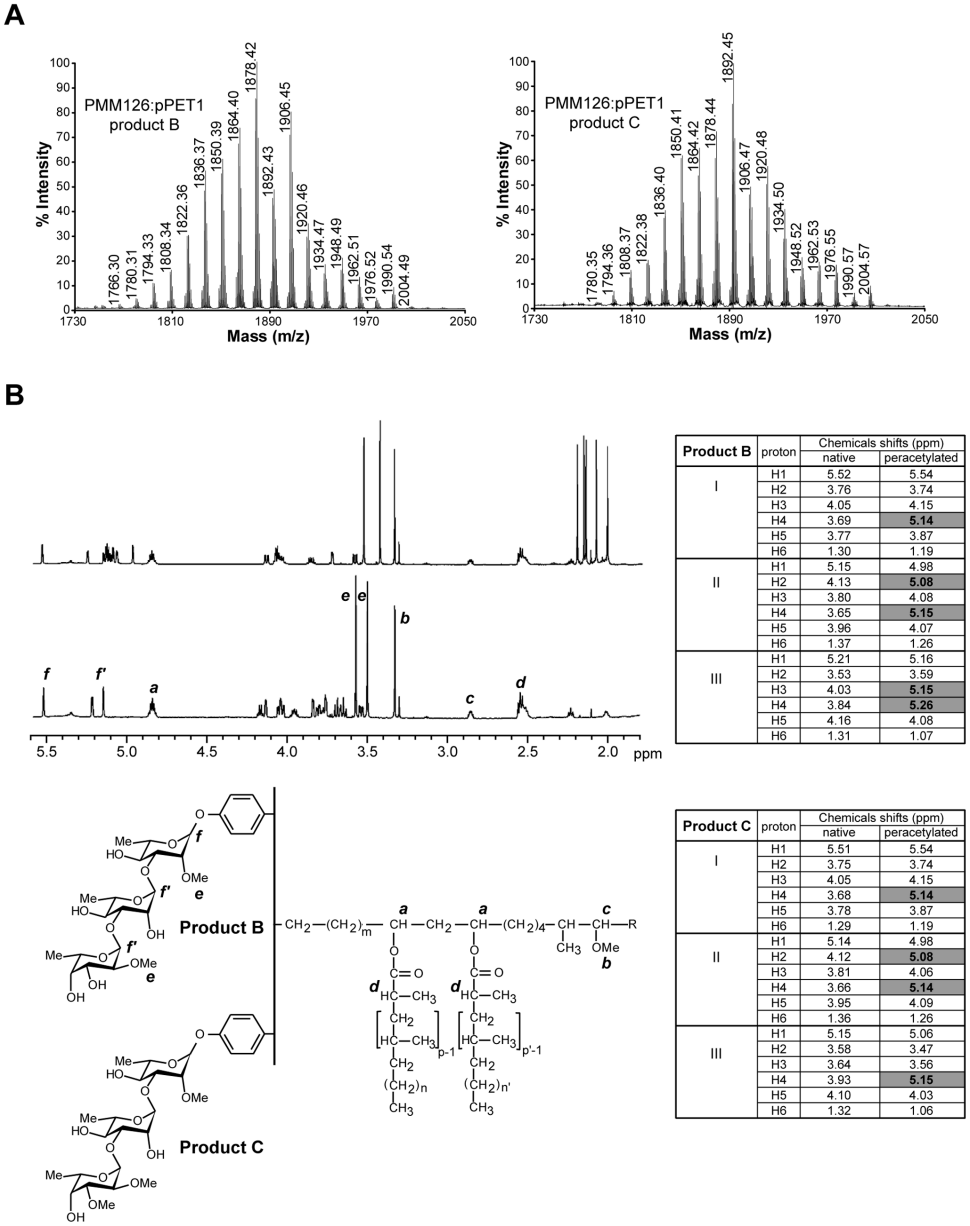
Biochemical analysis of products B and C from the PMM126:pPET1 mutant. (**A**) MALDI-TOF mass spectra of purified product B (left panel) and of purified product C (right panel) from the PMM126:pPET1 mutant strain**.** (**B**) NMR analysis of products B and C. 1D ^1^H-NMR spectra (1.8–5.6 ppm) of native (bottom) or per-*O*-acetylated (upper) glycolipid B (600 MHz, in CDCl_3_). The structures of the analyzed compounds are shown below the spectrum and the protons corresponding to the main signals are indicated. The tables summarize the assignments of resonances on the basis of chemical shift correlations deduced from the 2D-COSY spectra of native and per-*O*-acetylated compounds B and C. Proton resonances shifted by acetylation are written in bold (grey cells). The first column of each table indicates the sugar residue: I, first rhamnosyl residue; II, second rhamnosyl residue; III, terminal fucosyl residue.

The structures of products B and C, which accumulated in the *M. tuberculosi*s PMM126:pPET1 mutant were solved by both MALDI-TOF mass spectrometry and ^1^H MNR analyses of the purified compounds. The mass peaks from the major (product B) and from the minor (product C) products were, respectively, 28 and 14 mass units lower than those of PGL-tb from the H37Rv:pPET1 strain (Figure 5A) suggesting that the PMM126:pPET1 mutant produced two PGL-like substances that may differ from PGL-tb by the absence of two and one methyl group(s), respectively. All the proton resonances typifying a phenolphthiocerol structure linked to a trisaccharide were observed in the 1D ^1^H-NMR spectra of native compounds B (Figure 5B) and C (data not shown). Importantly, only two and three signals attributable to proton resonances of methoxyl groups linked to the sugar part (3.4–3.7 ppm) were observed in the spectra of native compound B (Figure 5B) and C (data not shown), respectively. This was in agreement with the presence of five and four signals of distinct methyl groups of acetyl substituents observed in the spectra of per-*O*-acetylated compounds B (Figure 5B) and C (data not shown), respectively, indicating the loss, compared to PGL-tb, of two and one methoxyl groups in the native compounds B and C, respectively. The positions of these free hydroxyl groups were assigned by 2D-COSY spectroscopy of native and per-*O*-acetylated compounds B and C (Tables in Figure 5B). The resonances of the protons 3 and 4 of the fucosyl residue were shifted after per-*O*-acetylation, indicating that the hydroxyl groups in positions 3 and 4 were free in the compound B. Likewise, it was possible to establish that position 4 of the fucosyl residue was the free hydroxyl group in the compound C.

These data demonstrate that *Rv2955c* is involved in the *O*-methylation at position 4 of the fucosyl residue of PGL-tb. However the accumulation in the PMM126:pPET1 strain of a major glycolipid that differs from PGL-tb by the absence of two methyl groups (product B) was surprising because *Rv2955c* encodes an enzyme that was expected to methylate the fucosyl residue at only one position. One explanation could be that disruption of *Rv2955c* in *M. tuberculosis* exerts a partial polar effect on the expression of *Rv2954c* involved in the *O*-methylation of position 3 of the fucosyl residue. Alternatively, it is possible that methylation of position 3 catalyzed by the product of *Rv2954c* occurs only after the *O*-methylation of position 4 by the methyltransferase encoded by *Rv2955c*. To discriminate between these hypotheses, we generated a new recombinant strain, named PMM145, with an unmarked mutation in *Rv2955c* (Figure S1) and we performed genetic complementation studies with plasmids pPET1 and pRS19 that contains a wild-type allele of *Rv2955c*. The PMM145:pPET1 strain synthetized two glycolipids that correspond to products B and C previously characterized in the lipids from the PMM126:pPET1 mutant (Figure 3 and Figure S2). Introduction of *Rv2955c* in the PMM145:pPET1 mutant restored the biosynthesis of a major glycoconjugate, which exhibits TLC mobility identical to that of PGL-tb (Figure 3). The MALDI-TOF mass spectrum of this lipid showed a series of pseudomolecular ion (M+Na^+^) peaks at *m/z* values identical to those observed in the mass spectrum of PGL-tb purified from the wild-type strain (Figure S2). Since the introduction of *Rv2955c* in the mutant strain was sufficient to restore the wild-type phenotype, we concluded that the disruption of *Rv2955c* in *M. tuberculosis* did not exert a polar effect on the expression of *Rv2954c*. These findings strongly support the conclusion that i) the product of *Rv2955c* is responsible for the *O*-methylation of the hydroxyl group located at position 4 of the fucosyl residue of PGL-tb and ii) the *O*-methylation of position 3 is dependent on the *O*-methylation of position 4 by the product of *Rv2955c*.

### Rv2956 Catalyses the *O*-methylation of Position 2 of the Fucosyl Residue of PGL-tb

**Figure 6:**
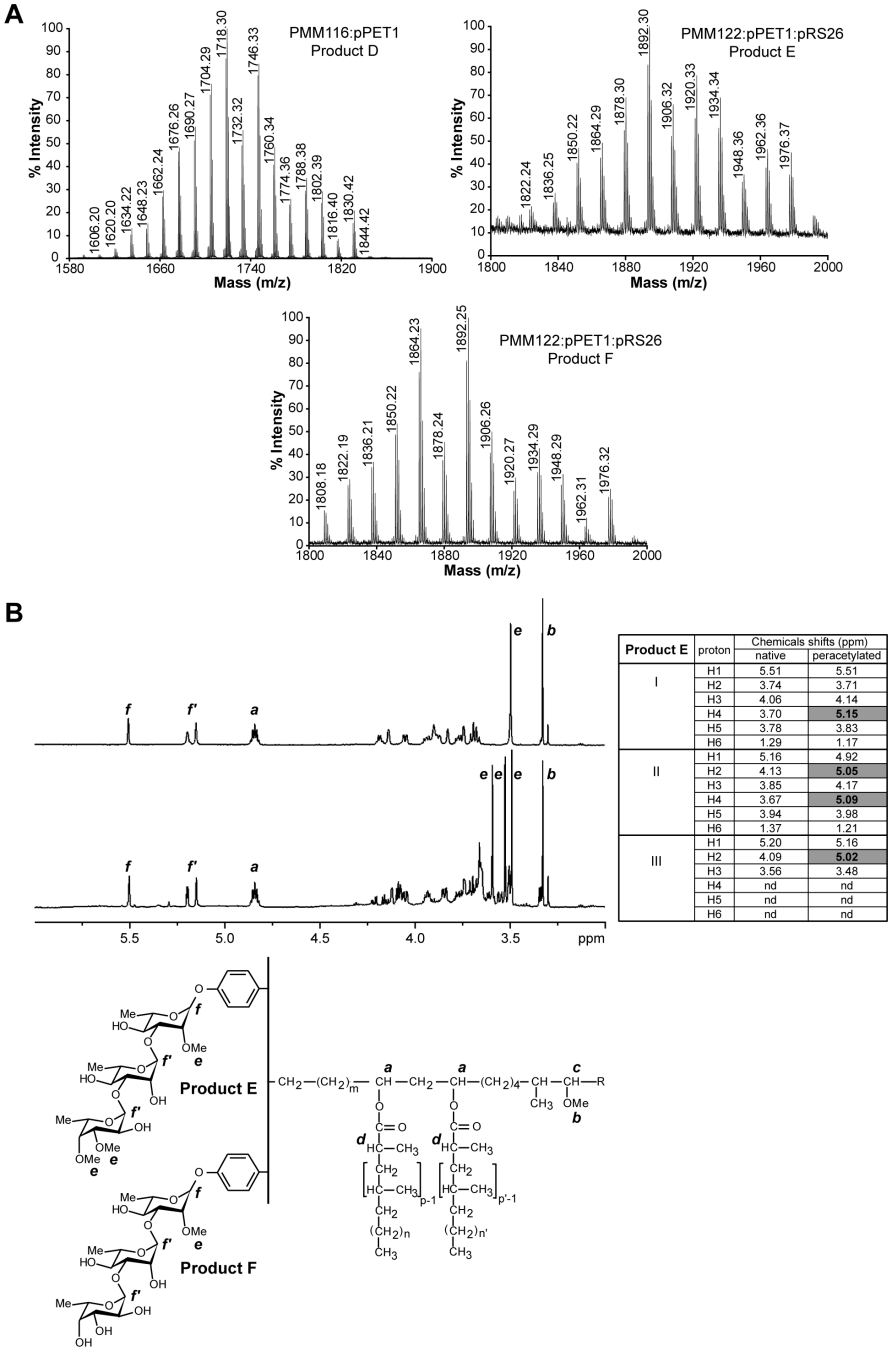
Biochemical analysis of products D, E and F. (**A**) MALDI-TOF mass spectra of purified product D from the PMM116:pPET1 mutant strain and of purified products E and F from the PMM122:pPET1:pRS26 mutant strain. (**B**) NMR analysis of products E and F. 1D ^1^H-NMR spectra (3.0–6.0 ppm) of native glycolipids E (bottom) and F (upper) (600 MHz, in CDCl_3_). The structures of the analyzed compounds are shown below the spectrum and the protons corresponding to the main signals are indicated. The table summarizes the assignments of resonances on the basis of chemical shift correlations deduced from the 2D-COSY spectra of native and per-*O*-acetylated compound E. Proton resonances shifted by acetylation are written in bold (grey cells). The first column of the table indicates the sugar residue: I, first rhamnosyl residue; II, second rhamnosyl residue; III, terminal fucosyl residue.

Product D, which accumulated in the *M. tuberculosis* PMM116:pPET1 mutant, was analyzed by MALDI-TOF mass spectrometry. Surprisingly, the mass peaks from this glycoconjugate were 188 mass units lower than those of PGL-tb (Figure 6). This difference corresponded to the mass of the tri-*O*-methylfucosyl residue of PGL-tb, suggesting that product D was a diglycosylated glycolipid. We reasoned that the insertion of the *km* cassette within *Rv2956* may exert a polar effect on the expression of the downstream *Rv2957* gene that encodes a glycosyltransferase involved in the transfer of the fucosyl residue in PGL-tb [18] (Figure 1), making it impossible to determine the putative function of *Rv2956* in the methylation of PGL-tb. To circumvent this problem, we generated a mutant with an unmarked mutation in *Rv2956*. This strain, named PMM122 (*Rv2954c::res*), was transformed either with pPET1 or with both pPET1 and pRS26, a plasmid harbouring a wild-type copy of *Rv2957*. The PMM122:pPET1 strain produced a glycolipid that showed a mobility on TLC identical to that of compound D from PMM116:pPET1 (Figure 3). MALDI-TOF analyses confirmed that it was indeed the same lipid (data not shown). In contrast the PMM122:pPET1:pRS26 strain accumulated four glycoconjugates (Figure 3). The major one was identified as product D by MALDI-TOF mass spectrometry (Figure S2) indicating a partial complementation of the polar effect on *Rv2957* expression by the wild-type *Rv2957* gene carried by plasmid pRS26. The PMM122:pPET1:pRS26 strain also produced two new PGL-like compounds, named E and F, and a small amount of PGL-tb (Figure 3).

The structures of products E and F were solved by both MALDI-TOF mass spectrometry and NMR analyses following purification. MALDI-TOF mass spectrometry analyses indicated that products E and F differ from PGL-tb by the absence of one and three methyl group(s), respectively (Figure 6A). The 1D ^1^H-NMR spectra clearly demonstrate the presence of phenolphthiocerol in compounds E and F. The presence of three deshielded anomeric proton signals (5.1–5.6 ppm) confirmed the occurrence of a trisaccharide moiety in both glycolipids. Focusing on the signals attributable to proton resonances of methoxyl groups linked to the sugar units (3.4–3.7 ppm), only one signal was observed in the spectrum of product F (Figure 6B) attributable to the methoxyl group linked on position 2 of the rhamnosyl residue linked to the phenol ring. This clearly indicates the loss in product F of the three methoxyl groups usually present on the PGL-tb fucosyl residue. For product E, three methoxyl signal resonances were observed (Figure 6B), compared to PGL-tb, confirming the absence of one methoxyl group in this compound. The position of the resulting free hydroxyl group was then identified by 2D-COSY spectroscopy of native and per-*O*-acetylated product E (Table in Figure 6B). The resonance of the proton 2 of the fucosyl residue was shifted after per-*O*-acetylation, indicating that the hydroxyl group in position 2 was free in product E. The finding that products E and F were not methylated at position 2 of the fucosyl residue indicated that *Rv2956* is involved in the 2-*O*-methylation of the terminal fucosyl residue of PGL-tb. In addition accumulation of product F which lacks *O*-methylation on the fucosyl residue, suggested that Rv2955c and Rv2954c display partial activities when position 2 of the fucosyl residue is unmethylated.

The PMM122: pPET1 strain was also transformed with plasmid pRS27 carrying the wild-type allele of *Rv2957* and that of *Rv2956* to complement both the polar effect on the *Rv2957* expression and the deletion of *Rv2956*. The resulting strain exhibited two major glycolipids that were identified as product D and PGL-tb by TLC and MALDI-TOF analyses (Figure 3 and Figure S2). The presence of product D indicated that the *Rv2957* gene carried by pRS27 weakly complemented the polar effect on the expression of the *Rv2957* chromosomal allele as observed with pRS26. However, the absence of PGL-tb like substance lacking a methoxyl group at position 2 of the fucosyl residue and the accumulation of large amounts of PGL-tb confirmed that Rv2956 is indeed involved in the *O*-methylation at position 2 of PGL-tb. Thus from these data it can be conclude that i) *Rv2956 O*-methylates the position 2 of the terminal fucosyl residue of PGL-tb and ii) Rv2954c and Rv2955c exhibit higher catalytic activity in the presence of Rv2956.

## Discussion

In this study, we provide genetic and biochemical evidences that *Rv2954c*, *Rv2955c* and *Rv2956* encode enzymes capable of *O*-methylating the terminal residue of PGL-tb at the 3-, 4-, and 2-position, respectively. Indeed, disruption of *Rv2954c* and *Rv2955c* in *M. tuberculosis* results, respectively, in the production of a glycoconjugate with a fucosyl residue that is not *O*-methylated at position 3 and 4. Interestingly, the lack of a functional *Rv2955c* gene also prevents efficient *O*-methylation of position 3 in *M. tuberculosis*. This effect was not due to a polar effect on the expression of *Rv2954c* in the mutant strain since introduction of a wild-type allele of *Rv2955c* in the PMM126:pPET1 mutant was sufficient to fully restore the wild-type phenotype.

Disruption of *Rv2956* in *M. tuberculosis* led to the accumulation of a glycolipid lacking the terminal fucosyl residue (product D) suggesting a polar effect on the expression of *Rv2957*. Complementation of the PMM122:pPET1 strain with *Rv2957* or with both *Rv2956* and *Rv2957* partially suppressed the polar effect, likely due to a weak expression of *Rv2957* from plasmids pRS26 and pRS27. Interestingly, the PMM122:pPET1 strain complemented with *Rv2957* synthesized faint amounts of PGL-tb raising the possibility that an unidentified methyltransferase would partially fulfil the role of Rv2956. Rv2956 shares a high degree of sequence identity with the product of *Rv1513* (68.7% identity) in *M. tuberculosis* (Figure S3A). *Rv1513* is likely not required *per se* for the *O*-methylation of position 2 of the fucosyl residue because transfer of functional *Rv1511*, *Rv1512* and *Rv2958c* genes from *M. tuberculosis* in *M. bovis* BCG that naturally lacks an *Rv1513* ortholog, led to the formation of a glycolipid that is structurally identical to PGL-tb [11]. However in a genetic context where Rv2956 is absent, it is possible that Rv1513 could partially *O*-methylate the 2-position of the terminal fucosyl residue of PGL-tb in *M. tuberculosis.*

Accumulation of product E which contains a terminal 3,4-di-*O*-methyl-fucosyl residue in the PMM122:pPET1:pRS26 strain and its absence in the PMM122:pPET1 strain complemented with both *Rv2956* and *Rv2957* is consistent with the role of Rv2956 in the *O*-methylation of position 2. Moreover the presence of product F indicated that Rv2954c and Rv2955c have reduced enzymatic activities when the position 2 of the fucosyl residue is not *O*-methylated. Therefore, Rv2956 is likely to be the first methyltransferase that acts on the fucosyl residue of PGL-tb. Consistently the various PGL-like variants that accumulated in the *Rv2954c* and *Rv2955c* mutant strains were *O*-methylated on position 2.

Altogether these data suggest that *O*-methylation of the fucosyl residue of PGL-tb is a sequential process: the product of *Rv2956* catalyses the *O*-methylation of position 2, to yield a 2-*O*-Me-α-L-Fuc*p*(1→3)-α-L-Rha*p*(1→3)-2-*O*-Me-α-L-Rha*p*(1→)phenolphthiocerol dimycocerosates; this latter product is then sequentially methylated at position 4 by the *Rv2955c*-encoded enzyme and later on at position 3 by the product of *Rv2954c* to give PGL-tb. Sequential *O*-methylation of sugar residue has been described in other bacteria such as *Streptomyces olivaceus* and *M. smegmatis* [19], [20]. For instance *M. smegmatis* produces cell-wall-associated components, named glycopeptidolipids (GPLs) which contain a lipopeptide core that is modified with *O*-methylated rhamnosyl units and an *O*-acylated 6-deoxy talosyl residue [21]. The rhamnosyl residue of GPLs can be *O*-methylated with up to three methyl groups at positions 2, 3 and 4 by the methyltransferases Rmt2, Rmt3 and Rmt4 [20], [22]. It has been shown that *O*-methylation by Rmt3 at the C3 carbon of the rhamnose was necessary for subsequent methylation by the 4-*O* methyltransferase Rmt4 and the 2-*O*-methyltransferase Rmt2.

**Figure 7:**
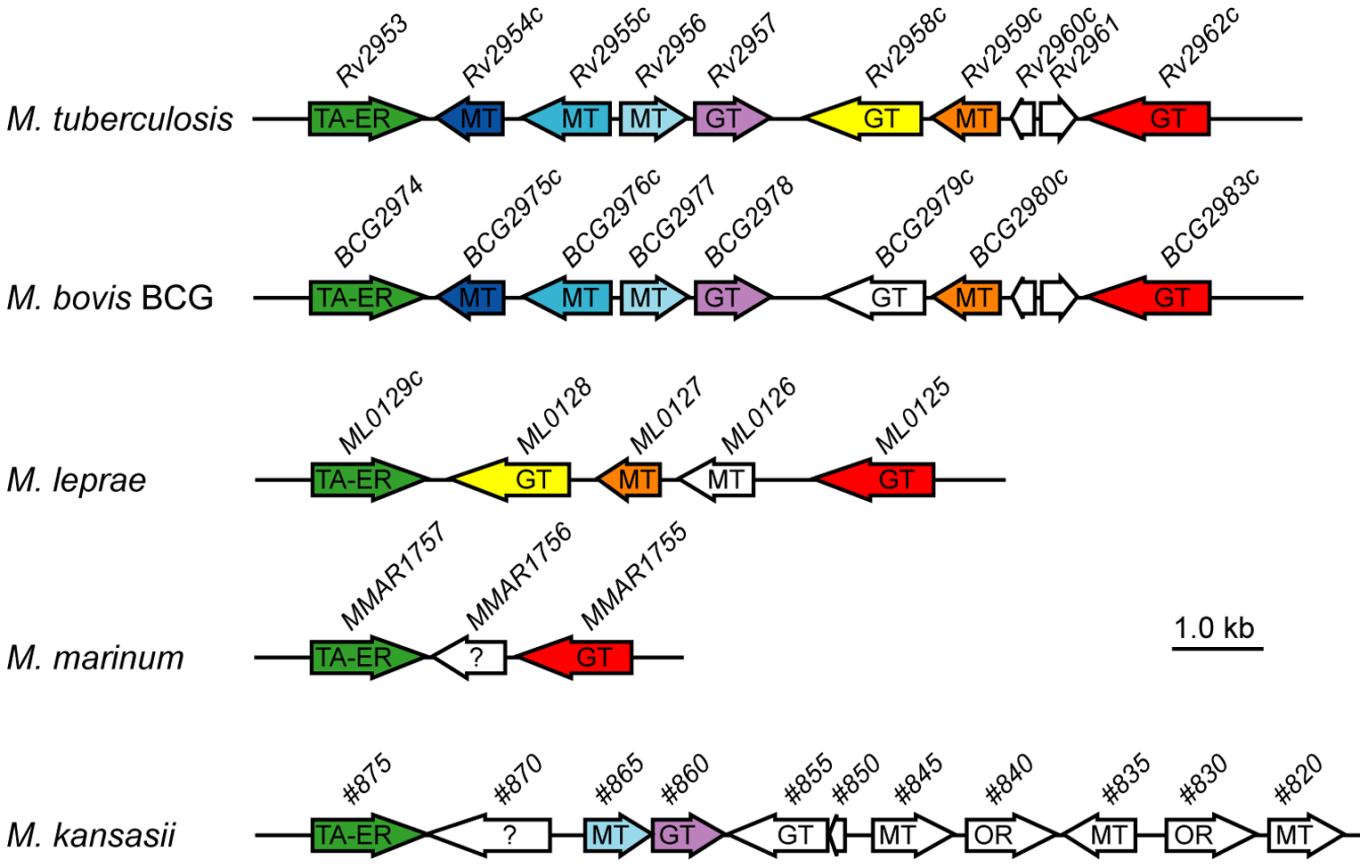
Genomic organisation of the DIM+PGL locus in *M. tuberculosis* from *Rv2953* to *Rv2962c* compared with the orthologous loci from *M. bovis* BCG, *M. leprae*, *M. marinum* and *M. kansasii*. The genes are represented by horizontal arrows and orthologous genes are shown by arrows of the same color. The enzymatic activities encoded by the various genes are indicated. TA-ER: trans-acting enoyl reductase, GT: glycosyltransferase, MT: methyltransferase, OR: oxydo-reductase. Question marks indicate that gene products have no assigned function. Genome organisation for *M. tuberculosis*, *M. bovis* BCG, *M. leprae*, *M. marinum* and ortholog predictions were obtained from the TubercuList, BCGList, Leproma, MarinoList databases available at the Pasteur institute web site (http://genolist.pasteur.fr). Genes are named according to the nomenclature used in these databases. Genome organisation for *M. kansasii* was obtained from the incomplete genome of *M. kansasii* ATCC 12478 available at xBase (http://www.xbase.ac.uk/genome/mycobacterium-kansasii-atcc-12478#). For brevity, gene names have been shortened (# means MkanA1_010100020, for instance #875 corresponds to MkanA1_010100020875). Ortholog predictions were obtained by comparing predicted protein sequence for each gene against the completed genome of *M. tuberculosis* using the BLAST program.

The proposed roles of *Rv2954c*, *Rv2955c* and *Rv2956* are consistent with the organization of the DIM+PGL chromosomal region in the various sequenced mycobacterial species producing PGL (Figure 7). Indeed conservation of these genes within the DIM+PGL locus correlates with the ability of these strains to synthesize a PGL containing an *O*-methylated fucosyl residue. For instance highly conserved *Rv2954c*, *Rv2955c* and *Rv2956* orthologs (*BCG2975c*, *BCG2976c* and *BCG2977*) are found in the DIM+PGL locus of *M. bovis* BCG. This mycobacterial species naturally produces a monoglycosylated PGL due to the lack of functional *Rv1511*, *Rv1512* and *Rv2958* orthologs; Consistently, transfer of these genes from *M. tuberculosis* in *M. bovis* BCG allows synthesis of PGL-tb in the resulting strain, indicating that *M. bovis* BCG possesses the enzymatic machinery necessary for the *O*-methylation of the fucosyl residue [11]. These reactions are most likely performed by the products of *BCG2975c*, *BCG2976c* and *BCG2977*. In contrast no *Rv2954c*, *Rv2955c* and *Rv2956* orthologs are present in the DIM+PGL loci of *M. marinum* and *M. leprae*, two mycobacterial species that produced PGL with no terminal *O*-methylated fucosyl residue (Figure 7) [2], [6]. Finally, the involvement of *Rv2956* in the *O*-methylation of position 2 of PGL-tb is supported by the genetic organisation of the DIM+PGL locus in *M. kansasii*. This chromosomal region contains several genes encoding putative *O*-methyltransferases but none of these display significant amino acid sequence homology to Rv2954c and Rv2955c (Figure 7). In contrast one gene encodes a protein exhibiting a high degree of sequence identity (about 84%) with Rv2956 (Figure S3B). Interestingly, the carbohydrate domain of the major form of PGL produced by *M. kansasii* consists of four sugar residues, the third one being a 2-*O*-Me-4-*O*-Ac fucosyl residue [2], [23].

The data presented in this study provide insights on the biosynthesis of compounds that are important for the pathogenesis of *M. tuberculosis*. The generation of mutants producing PGL-tb derivatives, and thereby *p*-HBAD derivatives, with a modified carbohydrate moiety could be useful tools to decipher the mechanisms by which these compounds act in the course of infection.

## Supporting Information

Figure S1 **Construction of the** ***M. tuberculosis*** **H37Rv** Δ***Rv2954c*** **,** Δ***Rv2955c*** **and** Δ***Rv2956*** **mutant strains.** Schematic diagram of the genomic organization of the *Rv2954c* (**A**), *Rv2955c* (**B**), and *Rv2956* (**C**) loci in the wild-type strain of *M. tuberculosis* H37Rv and in the various mutant strains generated in this study. The black boxes represent the *Rv2954c*, *Rv2955c*, *Rv2956*, and *Rv2957* genes. The *km*-resistance cassette used for targeted disruption and the two *res* sites are respectively represented by a light grey box and by dark grey boxes. Positions and names of primers used for the construction and screening of the mutant strains are indicated by arrows below each genetic structure and the expected sizes for PCR products are indicated. kb, kilobase.
(TIF)

Figure S2 **MALDI-TOF Mass Spectrometry Analyses.** (**A**) MALDI-TOF mass spectra of purified product A from the PMM144:pPET1 mutant strain and of purified PGL-tb from the PMM144:pPET1:pRS18 mutant strain. (**B**) MALDI-TOF mass spectra of purified products B and C from the PMM145:pPET1 mutant strain and of purified PGL-tb from the PMM145:pPET1:pRS19 mutant strain. (**C**) MALDI-TOF mass spectra of purified product D from the PMM122:pPET1:pRS26 and PMM122:pPET1:pRS27 mutant strains and of purified PGL-tb from the PMM122:pPET1:pRS27 mutant strain.
(TIF)

Figure S3 **Protein sequence alignments.** (**A**) Global alignment of *Rv2956* and *Rv1513* from *M. tuberculosis.* (**B**) Global alignment of *Rv2956* from *M. tuberculosis* and MkanA1_010100020865 (#: MkanA1_010100020) from *M. kansasii*. Alignments were generated using the LALIGN program (matrix file: BLOSUM50, gap open/ext: −14/−4) available at the SwissEMBnet web server (http://www.ch.embnet.org). “:” indicates identical residues in the aligned sequences and “.” indicates similar amino acid residues.
(TIF)
