# Supplementary figures and images for: Functional Characterisation of Three O-methyltransferases Involved in the Biosynthesis of Phenolglycolipids in Mycobacterium tuberculosis
Source: PLoS One. 2013 Mar 11;8(3):e58954. doi: 10.1371/journal.pone.0058954 (PMC3594219; doi:10.1371/journal.pone.0058954)

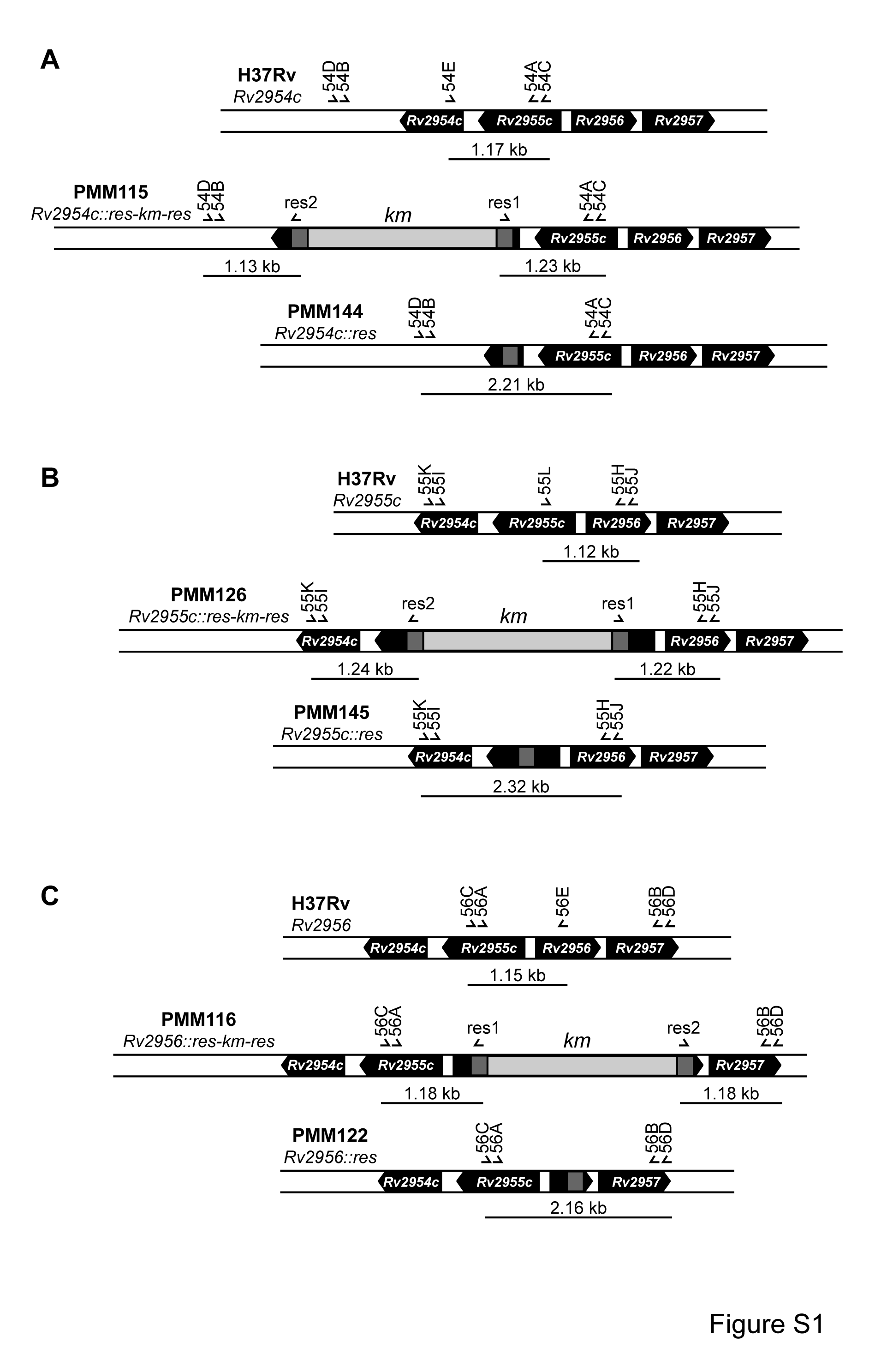

Supplement: Figure S1 — Construction of the M. tuberculosis H37Rv ΔRv2954c , ΔRv2955c and ΔRv2956 mutant strains. Schematic diagram of the genomic organization of the Rv2954c (A), Rv2955c (B), and Rv2956 (C) loci in the wild-type strain of M. tuberculosis H37Rv and in the various mutant strains generated in this study. The black boxes represent the Rv2954c, Rv2955c, Rv2956, and Rv2957 genes. The km-resistance cassette used for targeted disruption and the two res sites are respectively represented by a light grey box and by dark grey boxes. Positions and names of primers used for the construction and screening of the mutant strains are indicated by arrows below each genetic structure and the expected sizes for PCR products are indicated. kb, kilobase. (TIF) [file pone.0058954.s001.tif]

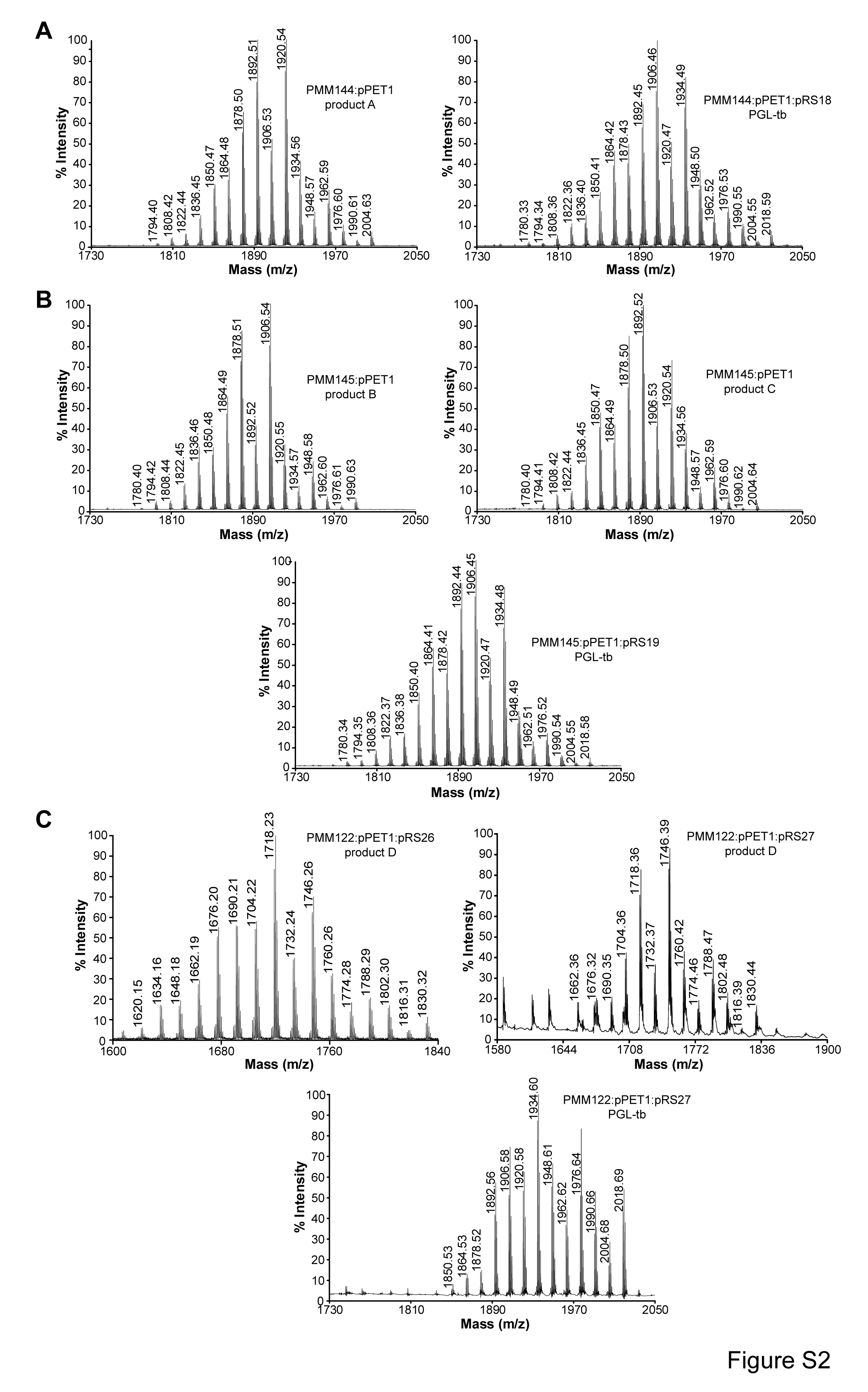

Supplement: Figure S2 — MALDI-TOF Mass Spectrometry Analyses. (A) MALDI-TOF mass spectra of purified product A from the PMM144:pPET1 mutant strain and of purified PGL-tb from the PMM144:pPET1:pRS18 mutant strain. (B) MALDI-TOF mass spectra of purified products B and C from the PMM145:pPET1 mutant strain and of purified PGL-tb from the PMM145:pPET1:pRS19 mutant strain. (C) MALDI-TOF mass spectra of purified product D from the PMM122:pPET1:pRS26 and PMM122:pPET1:pRS27 mutant strains and of purified PGL-tb from the PMM122:pPET1:pRS27 mutant strain. (TIF) [file pone.0058954.s002.tif]

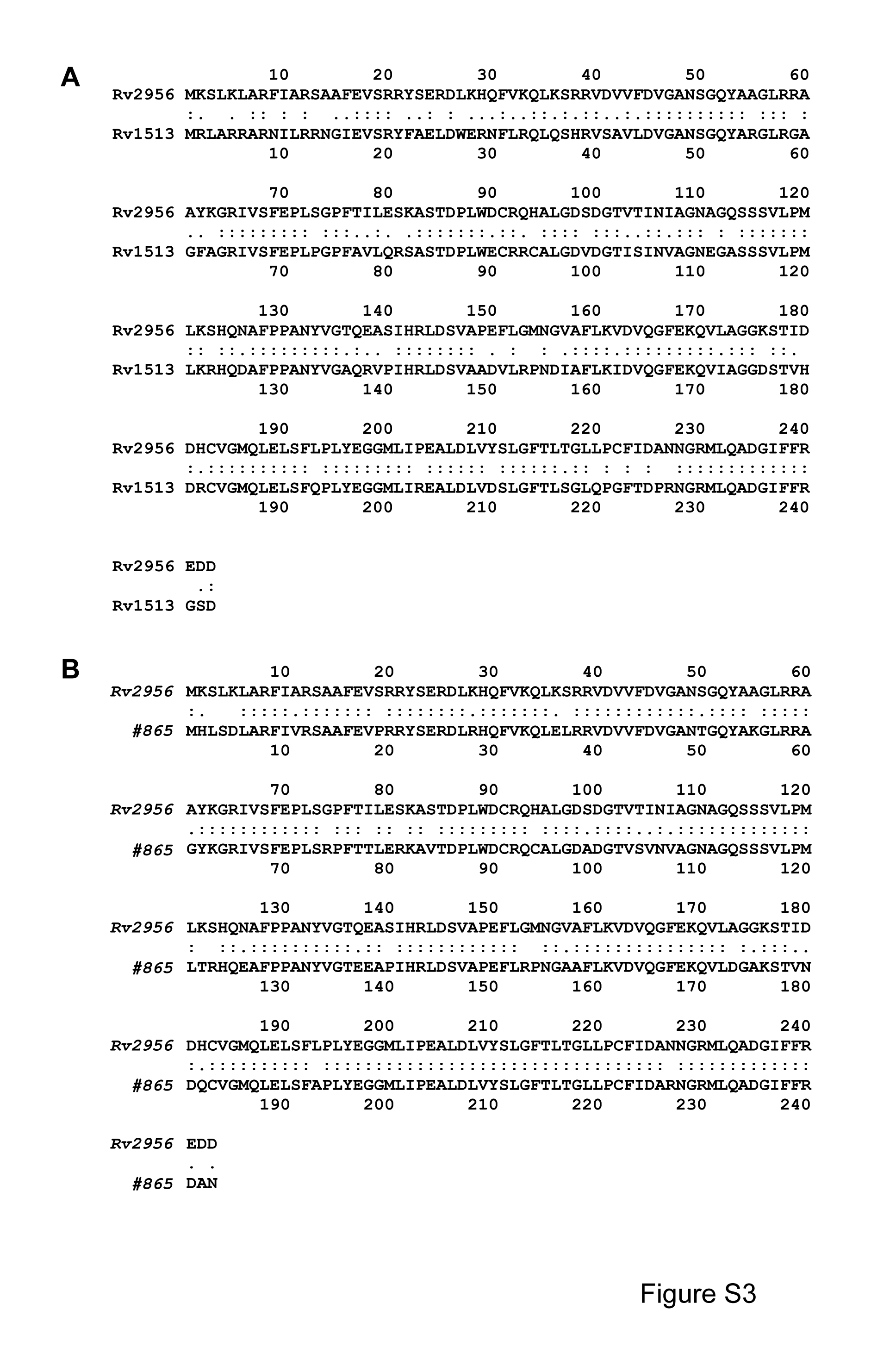

Supplement: Figure S3 — Protein sequence alignments. (A) Global alignment of Rv2956 and Rv1513 from M. tuberculosis. (B) Global alignment of Rv2956 from M. tuberculosis and MkanA1_010100020865 (#: MkanA1_010100020) from M. kansasii. Alignments were generated using the LALIGN program (matrix file: BLOSUM50, gap open/ext: −14/−4) available at the SwissEMBnet web server (http://www.ch.embnet.org). “:” indicates identical residues in the aligned sequences and “.” indicates similar amino acid residues. (TIF) [file pone.0058954.s003.tif]
